# Notes on Preparations for the Sick

**Published:** 1906-06

**Authors:** 


### Notes on Preparations for the Sick.

**Liq. Violæ Glucosidi** (Gadd).—Messrs. EVANS, GADD & Co., Exeter and Bristol.—With reference to the treatment of cancerous affections with violet leaves—a method now much in vogue—it is of interest to observe that in a paper read by Mr. H. Wippell Gadd, F.C.S., before the Therapeutical Society in December last, he gave particulars of a systematic examination of the leaves of the "Princess of Wales" violet. This may be summarised as follows:—

1. No volatile constituent was isolated.
2. No alkaloid could be detected.
3. No salicylic acid was found.
4. The presence of a glucoside was proved, and this reckoned as viola-quercitrin amounted to 5 per cent. of the weight of the fresh leaves.

A concentrated preparation, containing in each fluid part the glucoside present in one part by weight of the leaves, has been made, and the methods of making and standardising this were described in detail. We have used this fluid in two cases, and can state that it has no objectionable qualities, neither, so far as can be learnt from short experience, has it any positive merits.

**Alexine** (The Joulie Laboratory of Paris).—THOS. CHRISTY AND Co., London.—This preparation is made on the basis that phosphorus is necessary to the manifestation of brain power, and that the uncertain results of the known preparations have led to a temporary abandonment of what should be a most valuable drug. Alexine is believed to contain pure, free phosphoric acid; by association with biphosphate of manganese and biphosphate of iron, it is claimed that the whole quantity of phosphoric acid remains in an absolutely free state. It is a pale rose, granular powder, readily soluble in water. One to two teaspoonfuls are to be taken in water two or three times a day.

"**Sajodin.**"—THE BAYER Co., 19 St. Dunstan's Hill, London, E.C.—Under this name has been introduced the calcium salt of monoiodo-behenic acid. It is a colourless substance, containing about 25 per cent. of organically combined iodine, and is practically tasteless, thus possessing a distinct advantage over alkaline iodides. We have previously laid stress on the value of organically-combined halogen compounds, and clinical experiments have shown that "Sajodin" is no exception to the rule. It has been successfully administered in cases where the internal use of iodine was indicated, such as angina pectoris of vasomotor origin, in bronchial and in cardiac asthma. It is best prescribed in the form of powders, the dose being from 15 to 45 grains.

---

**Tabloid Xaxa**, gr. v.; **Tabloid Sodium Citrate**, gr. ii.; **Argyrol**, in tabloid and soloid; **Enule Gall and Opium**; **Tabloid Brand Pastilles.**—Burroughs, Wellcome & Co., London.—This firm does well whatever it undertakes, and the above preparations appear to be worthy of all confidence.

Tabloid Xaxa, gr. v.—"Xaxa" is a trade mark name to designate acetyl-salicylic acid. Acetyl-salicylic acid has been very widely prescribed, as it appears to possess all the valuable therapeutic properties of salicylic acid and its salts, without unpleasant after-effects. Acetyl-salicylic acid is insoluble in the stomach, but readily soluble in the intestine, and therefore causes no gastric irritation. It may be used in any conditions in which salicylic acid or sodium salicylate is usually employed. It is unquestionably of great service in gout and rheumatism, especially in connection with the ear affections associated with these conditions. It is also of conspicuous value in some forms of headache and neuralgia. It has been administered with success in chorea, pleurisy, glycosuria, eye diseases, and for the pains of tabes dorsalis and disseminated sclerosis. It has also been given for the relief of pain following abdominal operations, and may be employed generally as an analgesic. Acetyl-salicylic acid, presented as Tabloid Xaxa, readily disintegrates, and its therapeutic effect is promptly manifested. "Xaxa" contains no free salicylic acid.

One to three Tabloid Xaxa may be directed to be swallowed with water, plain or acidulated, twice or thrice daily, after food. For the relief of pain 15 grains may be prescribed as a first dose, with further doses if necessary of 10 grains at intervals of one hour to one hour and a half until three or four doses in all have been given.

Tabloid Sodium Citrate, gr. ii.—Difficulties associated with the digestion by infants of artificially-prepared foods frequently come under the notice of the medical practitioner. The necessary modification of cow's milk by dilution and sweetening to approximate human milk is often inefficiently carried out by the mother or nurse, consequently the infant may develop gastro-intestinal symptoms as a result of its inability to digest the food administered. The digestibility of cow's milk is greatly assisted by the addition of sodium citrate.

The explanation of the action which is commonly given is that the acid caseinogen and the calcium salts of milk in presence of the gastric juice form a thick casein clot. If sodium citrate be added to the milk it combines with the caseinogen to form a sodium compound less dense and more absorbable than the calcium caseinogen compound in the normal milk clot. The calcium salts in the milk unite with the citric acid of the sodium citrate, and the resultant calcium citrate is diluted by the stomach contents and absorbed. Thus the introduction of sodium citrate increases the digestibility of cow's milk in a remarkable manner, allows the absorption of the calcium salts, and greatly enhances the food value of the milk. The milk so modified is also a valuable antiscorbutic. This method of treating milk may be employed in cases where there is reason to believe that the nourishment of the infant is unsatisfactory, and is of great value in cases of poor patients who cannot carry out more complicated instructions regarding the modification of cow's milk. It is indicated when the mother's milk does not suit the child, and also in the process of weaning. It is used to enable the amount of milk given in twenty-four hours to be increased. It is valuable in arresting or avoiding milk dyspepsia.

The cheapness of this method of milk modification is a feature to be emphasised. It is directed that 1 to 2 grains or more of sodium citrate be given in each ounce of milk. One tabloid should be dissolved in about one teaspoonful of water and added as required to the milk. The tabloid should not be added directly to the milk.

ARGYROL.—Argyrol is an organic silver compound, strongly antiseptic, non-irritating, and non-toxic in the strengths usually employed. Five to 50 per cent. solutions are used in purulent and gonorrhœal ophthalmia and in lachrymal obstruction. In gonococcal urethritis initial injections of a 5 per cent. solution, gradually increased in strength, are recommended. Rectal injections of 1 to 5 pints of a 1 per cent. solution are employed in mucous and ulcerative colitis. Argyrol is stated not to precipitate albumin, and hence the solution penetrates the tissues without causing a superficial coagulum such as occurs with other agents. This property is of importance in gonorrhœa, the solution of Argyrol being capable of destroying gonococci in the deeper tissues. The following are issued:—

Tabloid Ophthalmic Argyrol, gr. $\frac{1}{24}$.—One placed directly on the conjunctiva dissolves in a few seconds.

Soloid Argyrol, gr. 1.—One dissolved in sufficient distilled water to produce a volume of 11 minims yields a 10 per cent. solution.

Soloid Argyrol, gr. 5.45.—One dissolved in 1 drachm of water yields a 10 per cent. solution.

ENULE GALL AND OPIUM.—Enule Gall and Opium presents an effective and convenient means of prescribing opium with the astringent principle of galls in the form of a suppository. Each contains extract of opium, gr. $\frac{1}{4}$, and tannic acid, gr. 3, equivalent to gr. 5 of galls. In hemorrhoids, rectal ulcer, fissure, congestion of the mucosa, rectal discharges, &c., the employment of this Enule should give good results.

TABLOID BRAND PASTILLES.—We have received several samples of these Tabloid Brand Pastilles. They present a series in which the utmost reliance may be placed. The agreeable demulcent basis is adjusted to dissolve slowly in the mouth, and so ensure the full efficacy of this method of administration. The list of Tabloid Brand Pastilles is as follows:—

1. Ammonium Chloride and Liquorice.
2. Benzoic Acid Compound—

| | | |
|---|---|---|
| ℞ | Acidi Benzoici ... ... ... ... ... | gr. $\frac{1}{2}$ |
| | Codeinæ ... ... ... ... ... ... | ,, $\frac{1}{10}$ |
| | Menthol ... ... ... ... ... ... | ,, $\frac{1}{10}$ |
| | P. Ipecacuanhæ .. ... ... ... ... | ,, $\frac{1}{10}$ |
| | Cocaine Hydrochloridi ... ... | ,, $\frac{1}{40}$ |
| | Gummi Rubri ... ... ... ... ... | ,, $\frac{1}{2}$ |

3. Cocaine Hydrochloride, gr. $\frac{1}{10}$.
4. Codeine, gr. $\frac{1}{8}$.
5. Glycerin.
6. Glycerin and Black Currant.
7. Glycerin, Tannin, and Black Currant.
8. Glycerin, Tannin, Capsicum, and Black Currant.
9. Heroin, gr. $\frac{1}{40}$, with Terpin Hydrate, gr. $\frac{1}{2}$.
10. Lemon Juice.
11. Linseed, Liquorice, and Chlorodyne.
12. Morphine and Ipecacuanha—

| | | |
|---|---|---|
| ℞ | Morphinæ Hydrochloridi ... | gr. $\frac{1}{36}$ |
| | Pulv. Ipecacuanhæ ... ... ... ... | ,, $\frac{1}{2}$ |

13. Pinol, min. 1.
14. Red Gum and Cocaine—

| | | |
|---|---|---|
| ℞ | Gummi Rubri ... ... ... ... ... | gr. 1 |
| | Cocainæ Hydrochloridi ... ... | ,, $\frac{1}{20}$ |

15. Rhatany, Menthol, and Cocaine—

| | | |
|---|---|---|
| ℞ | Ext. Krameriæ ... ... ... ... ... | gr. 2 |
| | Menthol ... ... ... ... ... ... | ,, $\frac{1}{20}$ |
| | Cocainæ Hydrochloridi ... ... | ,, $\frac{1}{20}$ |

16. Menthol, gr. $\frac{1}{8}$.

---

**Triferrin; Santyl.**—KNOLL & Co., 27a St. Mary-at-Hill, London, E.C.—Triferrin is a paranucleate of iron, composed of 22 per cent. of iron and $2\frac{1}{2}$ per cent. phosphorus, an organic combination readily soluble in a dilute solution of bicarbonate of soda, but not soluble in the hydrochloric acid of the gastric juice. It is conveniently prepared in tablets containing $4\frac{1}{2}$ grains of triferrin with some chocolate, also in a fluid form containing $1\frac{1}{2}$ per cent. of triferrin with aromatics, the compound being called Triferrol. The powder being free from taste is readily taken; it passes unchanged through the stomach, but is readily absorbed from the intestines, and should increase greatly the quantity of iron in the liver and the hæmoglobin in the blood. It cannot fail to be a very efficient method for the treatment of anæmic conditions; the dose is 5 grains.

Santyl (Knoll) is a tasteless, non-irritating salicylic ester of sandal wood oil. It may be given in doses of 30 drops in milk or in capsules. It is free from the unpleasant taste and smell of the usual preparations; it does not irritate either the intestinal or the renal tract, and is eminently efficient in urethritis and its usual complications.

---

We have received the following preparations from Messrs. Parke, Davis & Co., London:—**Acetozone,** antiseptic; **Acetozone,** inhalant; **Thyroidectin**; **Hypodermic Tablets,** Scopolamine and Morphine; **Mentholated Throat Tablets,** a compound of menthol and eucalyptol; **Acetanilide and Sodium Compound**; **Chocolate-coated Tablets**; **Gelatin-coated Evacuant Pill,** a compound of aloin, strychnine, belladonna and ipecac.; **Colchicine with Methyl Salicylate,** in globules; **Solution,** Iron Peptonate and Manganese.

Colchicine with Methyl Salicylate.—In globules, each globule consists of a soluble gelatin shell enclosing three minims of pure methyl salicylate in which is dissolved 1.250 grain of colchicine. The globules are made by a process that completely excludes air, yielding an attractive product that is easily swallowed and rapidly absorbed. The dose is one to two globules, three or four times daily.

Solution, Iron Peptonate and Manganese.—Solution of iron peptonate and manganese is entirely free from the usual objections urged against iron preparations. It is a semi-organic compound which, as soon as administered, is ready for immediate absorption by the intestinal villi.

The solution contains 0.6 per cent. of metallic iron and 0.1 per cent. of metallic manganese. The dose is one teaspoonful, to be taken three times a day, so that the patient receives not more than one grain of iron during the twenty-four hours. Much of the chalybeate medication of the past has proceeded upon the theory that the patient required to be dosed with large quantities of medicinal iron—a theory which holds good only when the absorption of the iron administered is extremely imperfect.

This is a very palatable fluid preparation of iron and manganese, and prompt results from its use may be expected. It is a valuable tonic in neurasthenia, convalescence, and general debility from any cause, also in the treatment of the anæmia of Bright's disease. It does not disturb the digestion or constipate the bowels.

Acetozone.—Under the name of "Acetozone," a valuable germicide has been placed upon the market. Chemically it consists of benzoyl-acetyl peroxide, a substance readily hydrolysed, the products of hydrolysis including benzoic acid and hydrogen peroxide, the latter body being a source of nascent Oxygen. Acetozone is recommended for the preparation of antiseptic lotions for various purposes, and also for internal administration. We consider the introduction of such an active preparation as the one described to be a distinct advance in the realm of germicides.

ACETOZONE INHALANT.—A solution of acetozone (1 per cent.) and chloretone (0.5 per cent.) in liquid paraffin. Valuable in all infectious or bacterial diseases of the nose, mouth, ear or throat, also in bronchitis and other inflammatory conditions of mucous membrane accessible by spray. It is practically non-irritating, and is best applied by means of the P. D. & Co. "Glaseptic Nebuliser."

THYROIDECTIN.—A powder prepared from the blood of animals which have been deprived of the thyroid gland. It was assumed that by such means thyroidism could be neutralised, and clinical tests have corroborated the theory. In the treatment of Graves's disease marked improvement has almost invariably been observed. Thyroidectin is non-toxic, and appears to be well borne by the stomach. It is supplied in capsules of 5 grains each, the dose being from 5 to 10 grains.

SCOPOLAMINE AND MORPHINE HYPODERMIC TABLETS.—Each contains 1.64 grain scopolamine hydrobromide and 2.5 grain morphine hydrochloride. This combination is recommended by Korff (*Berliner klinische Wochenschrift*) for the production of general anæsthesia or as an aid to ether or chloroform, which, if used at all, are needed in much less quantities than usual. One-third part of a freshly-prepared solution of one of these tablets is injected two hours and a half, one hour and a half, and half an hour before operating. Specially useful where ether or chloroform is contra-indicated. Reports state that salivation is not excited, that liability to nausea is lessened, and quiet and freedom from pain after operation are secured. (See *Brit. Med. Jour.*, Dec. 30th, 1905, p. 1703.)

---

**Validol**, a preventive and cure of sea-sickness.—ZIMMER & Co., Frankfurt-am-Main; 33 Lime Street, London.—Yet another remedy for sea-sickness. So many of these are in vogue, and are said to be so efficacious, that there seems to be little need for more. Validol is composed of menthol and valerianic acid, but, like all remedies of the kind, it can only be obtained from the firm which has given this name to a combination of unknown proportions. The one drug is said to quiet the stomach and the other the brain. One or two doses of 10 drops on a piece of sugar frequently prevents sea-sickness and always give relief. In the initial stages of sea-sickness (cerebral pressure, nauseous taste, salivation, giddiness, &c.) 10 to 15 drops of Validol on a piece of sugar should be taken, the patient then lying down for half an hour. Later on a glass of wine and a biscuit may be taken, whereupon, after the lapse of an hour or so, the patient as a rule feels fresh and quite able to partake of food. If necessary the simple treatment as described above is repeated.

---

**Droitwich Brine Crystals.**—WESTON AND WESTALL, LTD., 23 Botolph Lane, London, E.C.—We have received a specimen of these crystals, which are recommended as a home remedy for rheumatism. The curative effects of the Droitwich brine baths are too well known for comment, and the Brine Crystals are made from the natural water. Analysis shows the presence of chlorides and sulphates in combination with sodium and potassium, with some calcium and magnesium. The crystals are supplied in conveniently-sized bags containing 14 lb., this quantity being sufficient for the preparation of numerous baths.

---

**Farmhouse Bread.**—HENRY JONES, Broadmead, Bristol.—The products manufactured by Mr. Henry Jones have been before the public for many years, and have made an excellent reputation for themselves. We have recently made a personal trial of "Farmhouse Bread," and consider that it possesses the good qualities claimed by its maker.

